# Assessment of the Antimicrobial Potentiality and Functionality of *Lactobacillus plantarum* Strains Isolated from the Conventional Inner Mongolian Fermented Cheese Against Foodborne Pathogens

**DOI:** 10.3390/pathogens8020071

**Published:** 2019-05-21

**Authors:** Zafarullah Muhammad, Rabia Ramzan, Amro Abdelazez, Adnan Amjad, Muhammad Afzaal, Shanshan Zhang, Siyi Pan

**Affiliations:** 1 Key Laboratory of Environment Correlative Dietology, Ministry of Education, Huazhong Agricultural University, Wuhan 430070, China; shaunazss@163.com; 2 Food Biotechnology and Food Safety Laboratory, Huazhong Agricultural University, Wuhan 430070, China; rabiaramzan@webmail.hzau.edu.cn; 3 Department of Dairy Microbiology, Animal Producrion Research Institute, Agriculture Research Center Dokki, Giza 12618, Egypt; amorbiotic@yahoo.com; 4 Institute of Food Science and Nutrition, Bahauddin Zakariya University, Multan 60800, Pakistan; adnanft01@gmail.com; 5 Department of Food Science, Nutrition and Home Economics, Government College University, Faisalabad 54000, Pakistan; muhammadafzaal@gcuf.edu.pk

**Keywords:** *Lactobacillus plantarum*, foodborne pathogens, antimicrobial potency, antibacterial peptide, physico-chemical stability, conventional hard cheese

## Abstract

*Lactobacillus plantarum* are amongst the diversified lactic acid bacteria (LAB) species which are being utilized abundantly in the food industry. Numerous *L. plantarum* strains have been reported to produce several antimicrobial compounds. Diacetyl, hydrogen peroxide, organic acids, as well as bacteriocins can also be exemplified by a variable spectrum of actions. The current study was intended to conduct the screening and characterization of antimicrobial prospective of *L. plantarum* from traditional Inner Mongolian fermented hard cheese. Foodborne pathogens*, Salmonella typhimurium*, *Escherichia coli* O157:H7, *Listeria monocytogenes*, and *Staphylococcus aureus*, were examined by using the Oxford cup technique and the mixed culture inhibition assays. The resulting analyses disclosed that *L. plantarum* KLDS1.0344 indicated broad antimicrobial spectrum against all selected pathogens as compared to other LAB used in this study. Additionally, the decrement of the pathogen population was observed up to 3.47 logs in mixed culture inhibition assays. *L. plantarum* KLDS 1.0344 acid production was recorded up to 71.8 ± 3.59 °D in mixed culture while antimicrobial particles released in cell free supernatants demonstrated bacteriocin-like characteristics showing substantial pH stability (2.0–6.0), proteolytic enzyme reduced the antibacterial activity (15.2 ± 0.6 mm–20.4 ± 0.8 mm), heat stability (20 min at 120 °C) against selected pathogens. Moreover, the spectrum range of antimicrobial peptides after the partial purification was decreased as compared to the crude bacteriocin-like compound. The SDS-PAGE analysis showed the molecular weight range of partially purified bacteriocin from 12 to 45 kDa. After analyzing the obtained data from the current experimentation showed that the capability of *L. plantarum* KLDS 1.0344 to oppose the pathogen growth in vitro relies on the occurrence of organic acids along with bacteriocin-like compounds proving *L. plantarum* KLDS 1.0344 as a potentially appropriate candidate as an alternative bio-control agent against foodborne pathogens.

## 1. Introduction

The progressive attention of the clients towards natural and healthy diet has actuated the food science research and industry to explore and present natural compounds to process and preserve food products and to mitigate the utilization of chemical additives as antimicrobials. A careful and systematic research to control the Food Borne Diseases (FBDs) is a multi-faceted task obliging talents in the areas of food microbiology and chemistry, food control, food safety, as well as, food management [1,2,3].

Presently, various lines of research have been or being tried to encounter “the chemical problem” by “the natural solution”. Among these investigations, selection of the bacterial strains capable to develop compounds, which can be utilized as preservatives or antimicrobials, verified that the lactic acid bacteria (LAB) might be apposite microorganisms for such “natural solutions” [4,5,6].

Due to its technological and probiotic characteristics *Lactobacillus plantarum* is considered as the most important species of *Lactobacilli* [7,8]. The biosynthetical production of the bioactive peptides, enzyme systems, organic acids, exopolysaccharides, and vitamins are said to be one of the key mechanisms by which antioxidant, antimicrobial and probiotic activities are carried out [7]. The probiotic properties and antagonistic features of the *L. plantarum* strains could be the unique characteristics which enable them to be utilized as biocontrol agents against potentially dangerous microbes during processing and storage of the food and, it also elongates the shelf-life and safety of the fermented food products. The presence of these probiotic strains in fermented food systems can possibly contribute to the reduction of chemical compounds and can increase the health and wellbeing of the consumer [9]. The *L. plantarum* strains have been found to possess the best probiotic properties like acid and bile salt tolerance, the ability to adhere Caco-2 cells, the surface hydrophobicity properties, and significant hypochlesterolemic and antioxidant activities [10].

Various *L. plantarum* strains might have been identified to produce numerous antimicrobial agents against certain pathogenic microorganisms which are the main cause of food spoilage. This antimicrobial impact has frequently been attributed to the organic acid synthesis, like, lactic and phenyl lactic acids [11,12,13,14]. Conversely, the synthesis of bacteriocin-like or antimicrobial peptides has also been described to assert the antagonistic potentiality by the *lactobacilli* [15,16]. The hostility of certain bacteria and their extracellular substances present in cell free supernatants (CFS) provide useful prospects for food conservation [12,17]. Recently, due to having extensive ties with foods and the status of GRAS (generally recognized as safe) the LAB and their products have gained significant attention in the field of food processing and preservation [18]. As natural preservatives and ingredients for the starters to manufacture the probiotic functional foods, the LAB could be utilized to tackle the foodborne diseases. [19,20]. Hence, all this needs an obligatory selection of the *lactobacilli* strains and bifidobacteria with probiotic characteristics [21].

Isolation of *L. plantarum* strains have been carried out from different traditional cheese products, for instance several Iranian and Italian cheese varieties [22], Polish golka cheese [23], Greek Melichloro cheese [24], Turkish Karin Kaymak cheese [25], Serbian Zlatar cheese [26], Indian camel cheese [27], Brazilian ovine cheese [28], Tibetan Qula cheese [29], and West African soft cheese [30]. The key motive of the current assessment was to explore the antimicrobial potentials of *L. plantarum* spp., isolated from Hurood cheese, which is a conventional type of hard cheese. It contains 25% moisture content and it is traditionally manufactured and consumed in the Inner Mongolian region of China for hundreds of years and representing the natural antimicrobials against prominent foodborne pathogens [31]. About 121 strains of LAB have been isolated from Hurood cheese, amongst them seven isolates have been identified as *L. plantarum* and have been investigated for their probiotic potentials, including their adhering ability to Caco-2 cells, tolerance to acid and bile salt, in vitro cholesterol reducing capability, surface hydrophobicity, and antioxidant activities [7,10,32]. For the purpose, we screened out *L. plantarum* KLDS strains, 1.0317, 1.0318, 1.0344, 1.0386, 1.0628, 1.0985, 1.0986 from the customary fermented cheese in Inner Mongolia, against foodborne pathogens.

Pathogens used in our study were *Salmonella* spp., *Listeria monocytogenes Staphylococcus aureus* and *E. coli* O157:H7. Public health and quality of food products are greatly affected by the *Salmonella typhimurium*. It has been considered as the most common pathogen around the world causing foodborne illnesses [33]. For example only in China *Salmonella* species have been considered responsible for about 40% of bacteria related to food poisoning [34]. The majority of people infected with *Salmonella typhimurium* suffer from diarrhea, fever, abdominal cramps. Contaminated chicken, milk, cucumber, raw tuna, pork, beef, eggs and seafood are considered as the reservoir of *Salmonella typhimurium* and involved in the outbreaks of illnesses linked to these species [35,36]. *Listeria monocytogenes* also involved in several illnesses like abortion, gastrointestinal diseases and these diseases are related to foods supporting the proliferation of this bacteria. It may also cause meningoencephalitis with or without bacteremia and, more important, it causes intrauterine infection with high mortality [37,38]. Even the *Listeria monocytogenes* can survive in foods having water activity less than 0.85. These foods are termed as LMFs or low moisture foods like dry fermented sausages, cereals, tree nuts, fermented cheeses and infant formula powders [23,39]. *Staphylococcus aureus* can cause scarlet fever, respiratory diseases and life-threatening toxic shock syndrome as this syndrome involves the infections in nervous system, hematologic, renal, muscular and gastrointestinal systems [40,41]. Moreover, *Staphylococcus aureus* involves skin infections like folliculitis, furuncle and carbuncles. These skin illnesses are rarely curable which leads to life-threatening septicemia [42]. Food poisoning with symptoms like vomiting, diarrhea, dehydration and nausea can also be caused by *Staphylococcus aureus* [43]. Contaminated water, uncooked or partially processed foods like, juices, sprouts, leafy greens, ground beef, peanut butter, soy-nut butter and milk based fermented and non-fermented products are a rich source of *E. coli* O157:H7 [37]. *E. coli* O157:H7 contamination aggravates life-threatening hemolytic-uremic syndrome [44] and hemorrhagic colitis [45].

We have tested several *L. plantarum* KLDS strains to assess their potential against these foodborne pathogens. We have also explored the antimicrobial spectrum of *L. plantarum* KLDS strains, selected and used in this study. We studied the chemical characteristics of the molecules probably explaining the perceived antimicrobial potential, as well as, its stability.

## 2. Results

### 2.1. Antimicrobial Potentiality Screening by Oxford Cup Technique

Antimicrobial potentiality of *L. plantarum* strains against the given pathogens (foodborne) was evaluated by the Oxford Cup Technique. Subsequent data is presented in Table 1 and Table 2. *L. plantarum* (KLDS 1.0344) displayed most potent antimicrobial potential among all *L. plantarum* strains against the given pathogens. Specifically, the inhibition zone of *S. aureus* reached 15.8 ± 0.1 mm and 13.8 ± 0.0 mm by culture and CFS respectively. Whereas, the smallest inhibition zone was detected against *L. monocytogenes* by culture (9.2 ± 0.1 mm) and CFS (8.5 ± 0.0 mm). *L. plantarum* KLDS 1.0985 and its cell-free supernatants only displayed antimicrobial competency opposite to *L. monocytogenes* (8.8 ± 0.1 mm and 5.3 ± 0.1 mm successively) and *S.typhimurium* (4.9 ± 0.2 mm and 3.0 ± 0.1 mm), but no considerable effect detected against *S. aureus and E. coli* (Table 2).

*L. plantarum* KLDS (1.0318, 1.0317, 1.0344, 1.0386, 1.0985, 1.0986) altogether displayed antimicrobial ability counter to *L. monocytogenes,* but *L. plantarum* KLDS 1.0628 demonstrated antimicrobial activity (*p* < 0.05) against *S. aureus.* Moreover, their cultures produced larger inhibition zones compared to CFSs. The above-given results also indicate the part of antimicrobial potency of *L. plantarum* KLDS 1.0344 could be from microbes themselves. Therefore, in accordance with the obtained results from the screening of antimicrobial potentiality, *L. plantarum* KLDS 1.0344 was suggested to be the best efficient to inhibit pathogens (foodborne) and were utilized for further experimentations.

### 2.2. Determination of Inhibitory Substances of L. plantarum KLDS 1.0344 and Evaluate the Effect of Protease Enzymes on Their Activity

**Figure 1:**
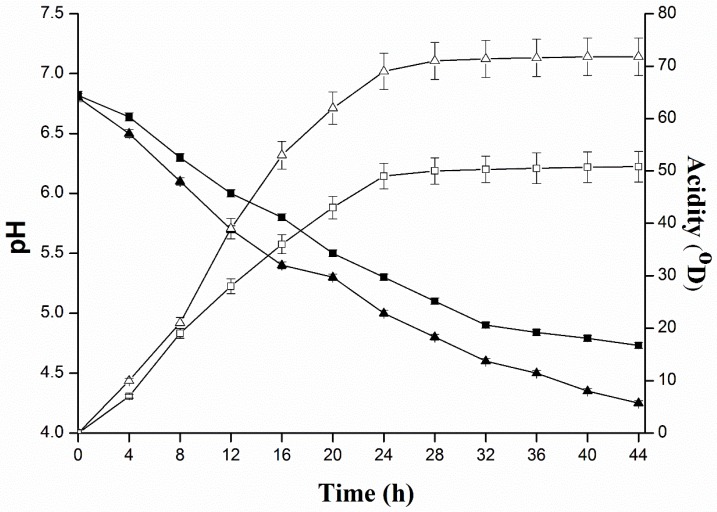
Evaluation of acidity (□ with solid line) and pH (■ with solid line) in pure culture and mixed culture of *L. plantarum* KLDS 1.0344, as well as acidity (△ with solid line and pH (▲with solid line) in mixed cultures of *L. monocytogenes* ATCC 19115, *S. typhimurium* ATCC 14028, *E. coli* O157:H7 ATCC 43889, *S. aureus* ATCC 25923.

The technological applicability of LAB characteristically grounded on the study of acidifying capability. In pure form, *L*. *plantarum* KLDS 1.0344 produced the highest levels of acidity 50.8 ± 2.94 °D in 44 h. In the mixed culture of *L. plantarum* with *S. typhimurium*, *L. monocytogenes*, *S. aureus* and *E. coli*, a high acidity (71.8 ± 3.59 °D) was witnessed at 44 h (significant *p* < 0.05). Kinetics of the pH progress (Figure 1) exhibited that *L*. *plantarum* reduced pH up to 4.7 ± 0.02 in 44 h, while, in the mixed culture of *L. plantarum* and *S. typhimurium*, *L. monocytogenes*, *S. aureus* and *E. coli* was 4.3 ± 0.01. Insofar as, an antibacterial behavior of bacteriocin-like composite from *L. plantarum* KLDS 1.0344 for all selected pathogens was significantly declined after treating with several different proteolytic enzymes such as, pepsin treated with 12.7 ± 0.5 mm for *L. monocytogenes*, 18.5 ± 0.8 mm for *S. aureus*, 16.3 ± 0.7 mm for *S. typhimurium*, and 15.6 ± 0.6 mm for *E. coli.*, From the results it was confirmed that the minimum inhibition zone was observed in bromelain enzyme case, 12.5 ± 0.5 mm for *L. monocytogenes*, 18.2 ± 0.7 mm for *S. aureus*, 16.0 ± 0.6 mm for *S. typhimurium*, and 15.3 ± 0.6 mm for *E. coli.* While the lowest decrease in inhibition zones were observed for protease treatment (Table 3). With every proteolytic enzyme treatment the antimicrobial activity clearly decreased, which exhibited that the antimicrobial compounds having a protein nature.

### 2.3. Partial Purification, Molecular Weight, Thermal and pH Stability of Bacteriocin

**Figure 2:**
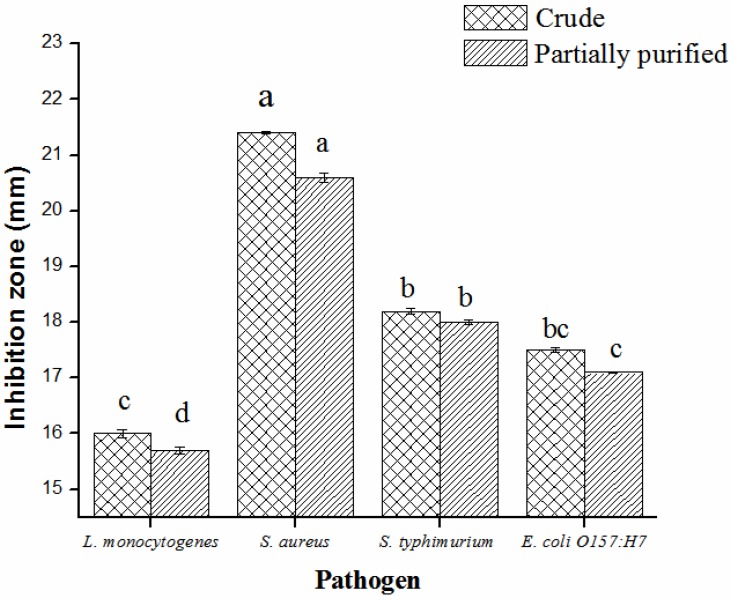
The antibacterial activities of the crude and partially purified bacteriocin isolated from *L. plantarum* KLDS 1.0344 against pathogens *L. monocytogenes*, *S. typhimurium*, *E. coli*, *S. aureus* measured by inhibition zone diameters. Bars signify the means of triplicate ± SD.

**Figure 3:**
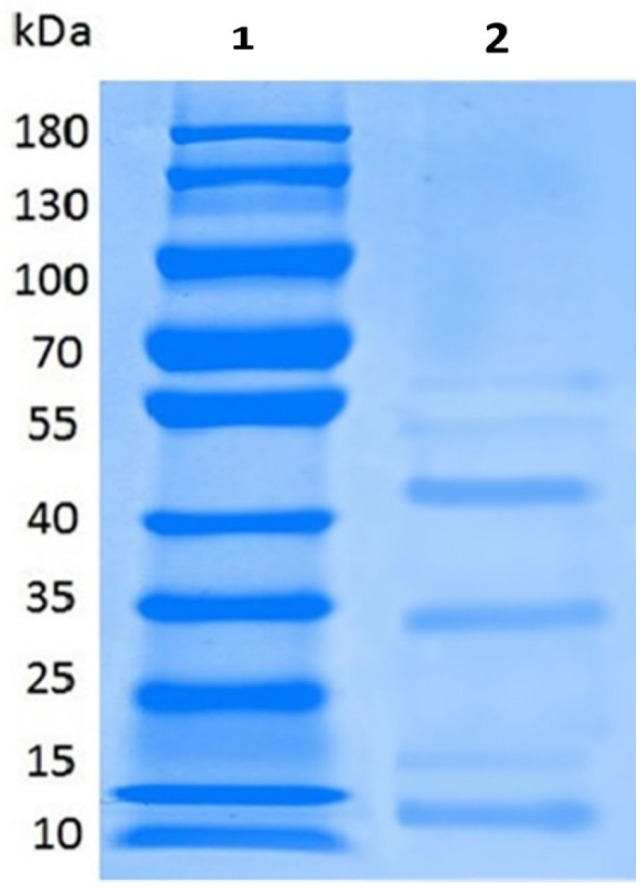
A bacteriocin protein profile of SDS-PAGE of *L. plantarum* 1.0344, Lane 1: Molecular weight marker; Lane 2: Partially purified bacteriocin protein produced by *L. plantarum* 1.0344.

In present research work, bacteriocins-like compounds, produced by *L. plantarum* (KLDS 1.0344), were partly cleaned by precipitation and dialysis of ammonium sulfate. Antimicrobial potential of bacteriocin was assessed against four pathogens, such as *S. aureus*, *S. typhimurium*, *E. coli* and *L. monocytogenes*. Inhibition zones of four pathogens were ominously dissimilar from each other after different treatments. Bacteriocin produced by *L. plantarum* KLDS 1.0344 after partial purification displayed ultimate antibacterial activity (16.0 ± 0.65–21.4 ± 0.83 mm) against the contestant pathogens as compared to the crude form (significantly *p* < 0.05) (Figure 2). The obtained partially purified bacteriocin from *L. plantarum* KLDS 1.0344 was treated at different levels of pH to assess its antibacterial potentiality against pathogens (Table 4). The antibacterial potentiality of peptides contrary to all pathogens was lessened with augmented pH values. No antibacterial activity was observed beyond a pH of 6.0. Hence, the pH range of antibacterial activities of obtained bacteriocin from *L. plantarum* KLDS1.0344 was pH 2–6. Table 5 also demonstrates that the thermal stability of bacteriocin resembling compounds were successively steady after subsequent treatments at 120 °C for 20 min retained inhibition activity of bacteriocin-like *L. plantarum* KLDS 1.0344 against pathogens *L. monocytogenes* (85.0 ± 0.7%), *S. aureus* (86.5 ± 0.3%), *S. typhimurium* (83.0 ± 0.0%), *E. coli* O157:H7 (81.0 ± 0.2%). According to the SDS–PAGE analysis, bacteriocins-like are small polypeptides with a molecular weight in the range of 12, 35 and 45 kDa (Figure 3).

### 2.4. Mixed Culture Inhibition Assay

**Figure 4:**
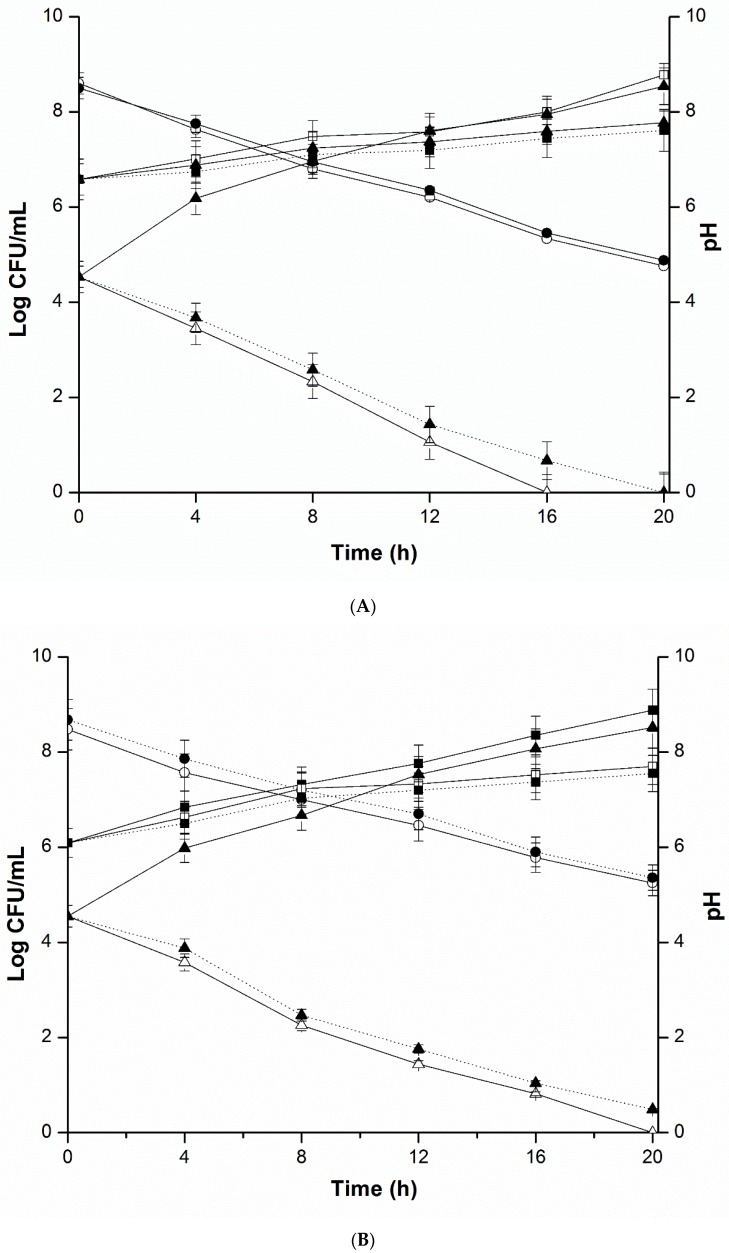

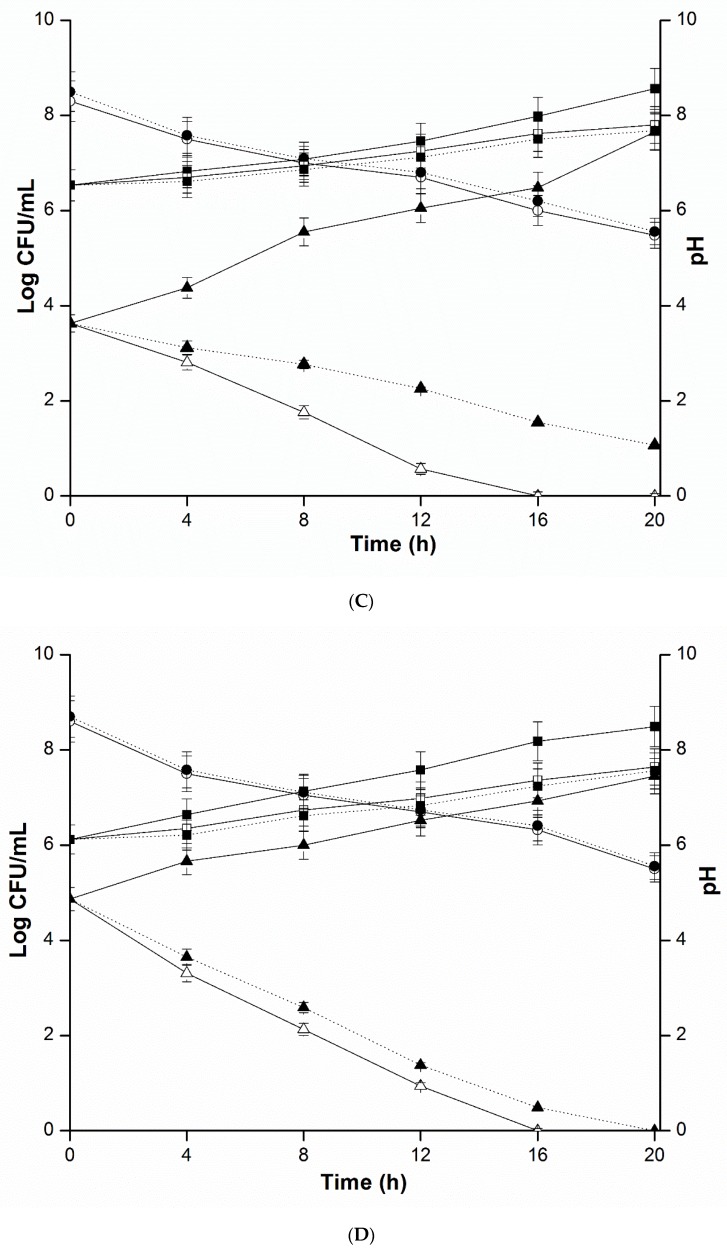
(**A**) Progression of *L. plantarum* KLDS 1.0344, pathogens and shift of pH in contact co-culture experiment (▲ with flecked line, ▲with solid line and △ with solid line) represents quantity of viable pathogens containing *S. aureus* in monoculture group, co-cultured with *L. plantarum* KLDS 1.0344 and diffusion chamber groups correspondingly; (■ with flecked line, ■ with solid line, and □ with solid line) represents viable counts of co-cultured with pathogens group, *L. plantarum* KLDS 1.0344 in monoculture group and diffusion chamber group correspondingly; (○ with solid line and ● with flecked line) represents pH of cultures in corresponding diffusion chamber and co-culture groups. (**B**) Progression of *L. plantarum* KLDS 1.0344, pathogens and shift of pH in contact co-culture experiment (▲ with flecked line, ▲with solid line and △ with solid line) represents quantity of viable pathogens containing *E. coli* O157:H7 in monoculture group, co-cultured with *L. plantarum* KLDS 1.0344 and diffusion chamber groups correspondingly; (■ with flecked line, ■ with solid line, and □ with solid line) represents viable counts of co-cultured with pathogens group, *L. plantarum* KLDS 1.0344 in monoculture group and diffusion chamber group correspondingly; (○ with solid line and ● with flecked line) represents pH of cultures in corresponding diffusion chamber and co-culture groups. (**C**) Progression of *L. plantarum* KLDS 1.0344, pathogens and shift of pH in contact co-culture experiment (▲ with flecked line, ▲with solid line and △ with solid line) represents quantity of viable pathogens containing *S. typhimurium* in monoculture group, co-cultured with *L. plantarum* KLDS 1.0344 and diffusion chamber groups correspondingly; (■ with flecked line, ■ with solid line, and □ with solid line) represents viable counts of co-cultured with pathogens group, *L. plantarum* KLDS 1.0344 in monoculture group and diffusion chamber group correspondingly; (○ with solid line and ● with flecked line) represents pH of cultures in corresponding diffusion chamber and co-culture groups. (**D**) Progression of *L. plantarum* KLDS 1.0344, pathogens and shift of pH in contact co-culture experiment (▲ with flecked line, ▲with solid line and △ with solid line) represents quantity of viable pathogens containing *L. monocytogenes* in monoculture group, co-cultured with *L. plantarum* KLDS 1.0344 and diffusion chamber groups correspondingly; (■ with flecked line, ■ with solid line, and □ with solid line) represents viable counts of co-cultured with pathogens group, *L. plantarum* KLDS 1.0344 in monoculture group and diffusion chamber group correspondingly; (○ with solid line and ● with flecked line) represents pH of cultures in corresponding diffusion chamber and co-culture groups.

While exploring the antimicrobial effects of *L. plantarum* KLDS 1.0344 on *E. coli*, *S. typhimurium*, *S. aureus* and *L. monocytogenes*, we executed the mixed culture inhibition assay and the results are shown in Figure 4. Observations revealed that in all groups pH of the cultures displayed evidential (*p* < 0.05) decline. Viable counts of *L. plantarum* with all pathogens in monoculture co-cultured and diffusion chamber groups are noticeably different (*p* < 0.05) among the groups with an increasing trend. Figure 4A–D) exhibits the growth of *L. plantarum* KLDS 1.0344 (maximum population of 8.8 log CFU per mL) was comparable to that attained in monoculture.

Whereas, decline in the number of viable cells of the *E. coli* O157:H7, *S. typhimurium*, *L. monocytogenes* and, *S. aureus* (ATCC 19115) in diffusion chamber, as well as in co-cultured groups ranged from 2.56 to 3.47 logs, were highly significantly different from the counts of all pathogen in monoculture (*p* < 0.001). Viable counts of pathogens decreased in diffusion chamber and co-cultured groups, whilst increased in monoculture group. Besides, the viable count of pathogens in the diffusion chamber group reduced up to 0 logs per ml after 20 h, while flatly declined to in co-culture group. Withal, in three groups the pH values of BHI were not significantly dissimilar (*p* > 0.05). Furthermore, the results from diffusion chamber culture and co-culture of *L. plantarum* KLDS 1.0344 with *E. coli*, *S. typhimurium*, *S. aureus* and *L. monocytogenes*, signifying the participation of a secreted inhibitory molecule from *L. plantarum* KLDS 1.0344, as well as, bacteria itself. However, only the antibacterial substances secreted by *L. plantarum* KLDS 1.0344 in the diffusion chamber group were involved in the inhibition of all pathogens. Bacterial cells were physically parted in the diffusion chamber group, while the diffusion of extracellular compounds and nutrients through the filter was probable. Whilst, contrasting the diffusion chamber group, bacterial cells could also interact with co-culture group pathogens. It might be stated that, the inhibitory effect of *L. plantarum* KLDS 1.0344 against the given foodborne pathogenic strains is due to the contact-dependent inhibition (CDI) mechanism.

## 3. Discussion

In the present study, *L. plantarum* KLDS 1.0344 (Table 1 and Table 2) displayed a stronger antimicrobial activity against the two pathogens (Gram positive) i.e., *S. aureus* and *L. monocytogenes*. And likewise, exhibited antimicrobial potentiality against two Gram negative pathogenic strains, i.e., *E. coli* and *S. typhimurium*.

Employment of LAB has been of significant interest to constrain the pathogens (*in vitro*). Presently, numerous *Lactococcus* and *Lactobacillus* species have been assessed for their executability to impede the progression of Gram negative and Gram positive infectious agents in different prototypical organisms [46]. The bactericidal effect of protease sensitive bacteriocins of the LAB has been mostly shown to obstruct the activities of Gram negative pathogens [47,48]. Conversely, the hostility of LAB against Gram-negative pathogens are thought to be owing to the formation of hydrogen peroxide and organic acids [49].

LAB comprising numerous *L. plantarum* strains had been investigated to yield varied antimicrobial agents, for instance, hydrogen peroxide, organic acids, bacteriocins, diacetyl and antimicrobial peptides, owing to a versatile range of mechanisms [17,50,51]. Some LAB, like, *L. plantarum*, display adverse effect towards spoilage and pathogenic microbes. This antagonistic attitude has been frequently attributed to produce (organic) lactic and phenyl lactic acids [12,13,14,52]. On the other hand, the production of (bacteriocins or bacteriocin-like) compounds has been found to explicate the antimicrobial potency applied by *lactobacilli* [15,16,53].

Numerous studies ascertained that LAB has the capability to yield antibacterial semantics counting, inhibitory enzymes, organic acids, diacetyl, bacteriocins, and hydrogen peroxide to constrain the growth of pathogenic strains up to a very wide range [54,55]. Loss of antibacterial activity of *L. plantarum* KLDS 1.0344 was found subsequently treating with pepsin, demonstrating that bacteriocin-identical compound was the peptide. After treating with protein digested enzymes like pronase, pepsin, bromelain, protease, ficin and α-chymotrypsin. Decreasing antimicrobial activity unveiled that the bacteriocin was peptide in nature (Table 3). Bacteriocins are the heterogeneously diverse group of proteins and antibacterial peptides that differ in their mode of action, the spectrum of activity, genetic origin, molecular mass and biochemical properties [56]. Some other researchers investigated the bactericidal influence might be due to the organic acids alone or in a blend with bacteriocins peptides [57].

Han et al. (2017) [58] and Katina et al. (2002) [59] stated that all *Lactobacillus* species retain eminent acidifying activity owing to the production of lactic and acidic acids. Anas et al. (2008) [60] ascertained that, after 24 h, the mixed culture of *L. plantarum* and *Staphylococcus aureus* produced a considerably excessive amount of lactic acid (69.4 °D).

After applying the proteases, it was perceived that the antibacterial agent of *L. plantarum* KLDS 1.0344 could be bacteriocin-like compound. Moreover, Pei et al. (2018) [61] investigated the antimicrobial potential of partly refined bacteriocins made by *Lactobacillus* sp. and displayed an impressive increase in antimicrobial effectiveness against *B. cereus*, *S. aureus* and *E. coli* (Figure 2). Bacteriocins have been demarcated as distinguished antimicrobial peptides/proteins, mainly directed to impede the growth of related species, detected in numerous genera of bacteria, including LAB [62].

Plantaricins are the bacteriocins manufactured by *L. plantarum* [63], while, Abo Amer, (2007) [64] witnessed the antimicrobial agent discharged by *L. plantarum* AA135 was greatly vigorous against a vast variety of Gram negative and Gram positive pathogens. Furthermore, Martine et al. (2013) [65], and Todorov et al. (2010) [66] reported that the bacteriocins like bacST216Ch and bacST202Ch, excreted by the *L. plantarum* strains sequestered from Chourico and Beloura, restrained the growth of certain meat putrefying Gram negative and positive bacteria. *L. plantarum* B0105 isolated from the conventionally fermented mustard of Taiwan, pragmatic to excrete bacteriocin that inhibited the *Streptococcus mutans* [67]. *L. plantarum* isolated from goat feta cheese exhibited bactericidal impact to resist *Listeria monocytogenes* [65].

Numerous *lactococci* and *lactobacilli* species are nominated to be inhibitory against *S. aureus* and *E. coli* in vivo and in vitro [36,60,68,69,70,71,72,73,74]. Lactic acid bacteria strains like *Lactobacillus plantarum, Lactobacillus paracasei and* four strains of *Lactobacillus fermentum*, not only, showed the better probiotic potential, but also, decreased the proliferation of enteropathogenic bacteria which were isolated from healthy infant feces, such as, *Shigella flexneri Yersinia enterocolitica*, *Salmonella enteritidis* and *Shigella sonnei* [75]. These *Lactobacillus* strains have been proved to be useful to treat diarrhea [76].

The complete disappearance of antibacterial properties of bacteriocin obtained from *L. plantarum* KLDS 1.0344 was detected against *S. aureus*, *S. typhimurium*, *E. coli.* and *L. monocytogenes* when pH was adjusted to 7 (Table 4). Lin et al. (2008) [57] also disclosed that the cultures of LAP5 cells neutralized to 7.0 pH, the alienating properties against *Salmonella* indicated non inhibitory potential. Alike findings testified that at pH 5.0, acidocin B produced by *L. acidophilus* retained 50% activity [77]. Main categories of LAB tailored bacteriocins consisted of lantibiotics, large heat-stable proteins and complex peptides, while heat stable property of bacteriocin compounds increased their applications [56,77]. In our present research work, the bacteriocin from *L. plantarum* KLDS 1.0344 has been found considerably heat and pH tolerant and potent bio preservative agent likewise. These potentials could make it useful natural food additive in food applications, which are at present, the matter of broad and extensive research. Searching the new bacteriocins encompassing a panoramic spectrum of antimicrobial activity are being investigated by some researchers.

Furthermore, results from Figure 4A–D) exhibited the viable count of four pathogens declined at 3.47 logs in the diffusion chamber group and 2.56 logs in co-culture group. These findings were analogous to the results of Atassi et al. (2006) [78] study, which disclosed that in viable cells of bacteria, the *L. helveticus* KS300 reduced pathogenic *E. coli* C1845, *S. typhimurium* SL1344 and IH11128 with a reduction of 2.0–5.5 logs. We should essentially emphasize that the unusual factors might be prospective for these outcomes and additional positive aspects could more decrease pathogenic progression. Former investigations on co-culture reticence provided an antimicrobial theory known as contact-dependent inhibition (CDI) mechanism [79,80,81]. In the present study it has been shown that the antimicrobial potential of the *L. plantarum* was associated with bacteria itself and bactericidal activity of the diffusion chamber group was compromising than the co-culture group. It is expected that, the antimicrobial action of bacteria itself could be correlated to the previously stated CDI mechanism. This mechanism could be illuminated by the interchange of information amongst the bacteria. Such transfer of information encompassed, secretion systems, conjugation, allolysis, and nanotubes and contact-dependent inhibition [79].

Type IV and VI secretion pathways were ascertained in (Gram negative) bacteria. They assist the transportation of molecules, proteins or DNA straight into prokaryote cells from the bacterial cytoplasm. Conjugation is a straight allocation of genetic material amongst the microbial cells by a bridge-like connection or through smooth cell-to-cell contact [82]. Apart from these pathways, several researchers also verified that certain *Escherichia coli* strains might cause CDI of other *E. coli* strains [81,83,84]. CDI mechanism was identified among proteo-bacteria, but did not demonstrate experimentally in (Gram positive) bacteria [85].

According to Jabbari et al. (2017) [86], during co-cultivation with the pathogens and separate cultivation at 37 ± 1 °C, *L. plantarum* kept the high concentration of viable cells. *L. plantarum* reduced the concentration of viable cells of *E. coli* ATCC 25922, *Klebsiella pneumonia* and *Salmonella* spp. The researcher has described thatthe inhibition of the growth of pathogens was attributable to generate other organic acids and lactic acid, which acidified the medium and altered the conditions for the growth of the pathogens.

## 4. Materials and Methods

### 4.1. Bacterial Strains and Growth Conditions

*L. plantarum* KLDS 1.0317, 1.0318, 1.0344, 1.0386, 1.0628, 1.0985, 1.0986 and other *Lactobacilli* utilized for antimicrobial spectrum were sequestrated from conventional cheese in Inner Mongolia and stored in Key Lab Dairy Science, Ministry of Education, China. 16S rDNA sequence analysis was used for the identification of these strains and these were anaerobically incubated in modified de Man, Rogosa, and Sharpe (mMRS) broth [87] at 37 °C. Four pathogenic strains, namely, *Escherichia coli* O157:H7 (ATCC 43889), *Salmonella typhimurium* (ATCC 14028), *Listeria monocytogenes* (ATCC 19115) and *Staphylococcus aureus* (ATCC 25923) were procured from entry-exit IQB (Inspection and Quarantine Bureau), Hubei province of China. These pathogenic strains were then aerobically incubated in BHI (Brain Heart Infusion broth, Qingdao Hope Biotechnology Co., Ltd. Shandong, China) at 37 °C and were utilized as indicator bacteria for the antimicrobial assays.

### 4.2. Preparation Cell Free Supernatants of L. plantarum and Screening for Antimicrobial Potentiality

2 mL of *L. plantarum* cultures (10^8^ CFUmL^−1^) were inoculated individually into mMRS broth (100 mL) and incubated for 24 h at 37 °C (Intelligent Biochemical Incubator, SPX-150B, Yangzhou, China). Then centrifugation of the cultures was done for 10 min at 10,000× *g* and 4 °C (Thermo Sorvall Legend Micro, 21 Microcentrifuge, ThermoFisher, Waltham, MA USA). Supernatants were then filter-sterilized through sterile filters 0.22 μm-pore-sized (Tianjin Navigator Lab instrument Co., Ltd. Tianjin, China) after discarding the bacteria precipitate.

The Oxford cup technique for the antimicrobial potentiality of *L. plantarum* KLDS strains against foodborne pathogens was explored in accordance to Wang et al. 2007:2009 [88,89] and Zhai et al. (2015) [90], with some adjustments. Initially, 12 mL of agar medium (1.5% *w*/*v*) was poured into the plate and was solidified. Formerly, in the stationary phase, 1% of indicator strain was injected into 12 mL of apposite BHI agar 1.2% (*w*/*v*), at 45 °C. Then, the mixtures were moved onto agar media and permitted to harden. Then, three sterilized Oxford Cups were taken. These cups were placed and pressed slightly on BHI agar surface, to degas the interspace between agar surface and cups. Later on, 200 μL of CFS and the same volume of culture dribbled into two cups, respectively. Then, as control, sterile water (200 μL) delivered into the third cup. Plates were piled into incubator by providing aerobic conditions at 37 °C for 24 h or 48 h and, antimicrobial activity was witnessed around the Oxford Cups, close to growth-free inhibition zones. Inhibition zones computed from the edges of cups in mm. The current trial was performed in triplicate.

### 4.3. Antibacterial Ambit of L. plantarum KLDS 1.0344

The antibacterial ambit of *L. plantarum* KLDS 1.0344 was evaluated in comparison to eleven indicator strains enclosing *Lactobacilli* and foodborne pathogens by the use of Oxford Cups. Primarily, 12 mL of agar medium (1.5% (*w*/*v*) was dispensed into the plate and uphold till solidified. In the stationary phase, 1% of indicator strain was injected into 12 mL of apposite agar medium 1.2% (*w*/*v*), at 45 °C. Formerly on agar medium the mix transferred and permitted to congeal. Then, three sterilized Oxford Cups were taken. These cups were placed and pressed slightly on BHI agar surface, to degas the interspace between agar surface and cups. Later on, 200 μL of CFS and the same volume of culture dribbled into two cups, respectively. Then, as control, sterile water (200 μL) delivered into the third cup. Plates were piled into incubator by providing aerobic conditions at 37 °C for 24 h or 48 h and, antimicrobial activity was witnessed around the Oxford Cups, close to growth-free inhibition zones. Inhibition zones computed from the edges of cups in mm. The current trial was performed in triplicate.

The plates were put in incubator providing aerobic conditions at 37 °C for 24 h or 48 h and around the Oxford Cups. Inhibition zones computed from the edges of cups in mm. The incumbent experiment conceded in triplicate.

### 4.4. Quantification of Acid Production of L. plantarum KLDS 1.0344

To determine the acid production capability of *L. plantarum* KLDS 1.0344, a deduction of 10 mL of pure culture *L. plantarum* KLDS1.0344 and mixed culture with *E. coli, L. monocytogenes*, *S. typhimurium* and *S. aureus* was individually transferred in a 100 mL conical flask and 5 droplets of phenolphthalein indicator (2 mg/mL in ethanol 60°) were imparted. NaOH 1/9N was used to neutralize acidity until the appearance of a persistent pink color. Titrating solution volume was deliberated to prefigure the production of acidity, assessed in dornic degree and a PHS-3C electrode pH meter (METTLER TOLEDO, Switzerland) was used to measure the pH of the culture after every 4 h. Each experiment was replicated thrice [60].

### 4.5. Mixed Culture Inhibition Assay

Antimicrobial potentiality against *E. coli*, *S. aureus*, *S. typhimurium* and *L. monocytogenes* cells was performed by using a mixed culture method. Equal volumes of *L. plantarum* KLDS1.0344 (10^6^ CFU per mL) and 10^3^ CFU per mL of each pathogen were co-cultured into a diffusion chamber which was split with a 0.22 μm size of the filter in their respective cultivating medium as described by Saraoui et al. (2016) [79] and, incubation was executed at 37 °C for 24 h. 1% of both *L. plantarum* KLDS 1.0344 and pathogens 10^6^ CFU per mL and 10^3^ CFU per mL, respectively were mono-cultured into mMRS and BHI broth used as a control medium. Every 4 h, all the cultures were dispersed onto mMRS agar and BHI plates with proper dilutions, and 20h incubation was done at 37 °C. Colonies of *L. plantarum* KLDS 1.0344 and pathogens were computed. PHS-3C electrode pH meter (METTLER TOLEDO, Switzerland) was used to measure the pH of above-mentioned cultures, after every 4 h. Every experiment was replicated thrice.

### 4.6. Antimicrobial Peptide Production

100 mL mMRS broth was inoculated with 2% *L. plantarum* KLDS 1.0344 (10^8^ CFU per mL) and incubated at 37 °C for 24 h. Afterwards, culture centrifugation was performed at 10,000× *g* for 10 min at 4 °C. All supernatants were then filter-sterilized using a sterile porous filter (0.22 μm). 1N NaOH was used to neutralize the resulting CFS to pH 6.5 and catalase (5 mg per mL) was used for the elimination of the hydrogen peroxide inhibitory effect. Finally, neutralized CFS (bacteriocin-like and bacteriocin metabolites) of the *L. plantarum* were tested against four pathogenic bacteria (*L. monocytogenes*, *S. aureus*, *E. coli* O157:H7 and *S. typhimurium*) to evaluate the antibacterial activity [91].

### 4.7. Antimicrobial Fractional Refinement by Dialysis and Ammonium Sulfate Precipitation

20 mL of crude bacteriocin-like were taken and ammonium sulfate solution (60%) was incrementally dispensed on the sample in a glass beaker. A magnetic stirrer (85-2 Hangzhou instrument motor Co., Ltd., Hangzhou, China) was used to stir the mixture for 2 h. Centrifugation (at 10,000 rpm) of the solution was accomplished at 4 °C, for 20 min. The precipitate was re-suspended in 25 mL buffer of potassium phosphate (10 mM, pH 7.0), after discarding the supernatant. Dialysis membrane was cut into the desired length, softly squeezed and opened. A cutoff value of the dialysis membrane was less than 1200 Da. The membrane was closed by fastening with a thread from one end. Samples of bacteriocin-like peptides were decanted on the dialysis membrane and knotted on a glass rod. Then, it was retained half soaked in (10 mM) phosphate buffer solution in a beaker and gently stirred for 12–18 h with a magnetic stirrer. To confirm appropriate dialysis of protein, the buffer was changed for every 3 h during the mixing procedure. Membrane bag was collected after dialysis completion, and stored at 4 °C carefully. Finally, the determination of the antibacterial activity of the dialyzed bacteriocin was performed by agar well diffusion procedure [91].

#### 4.7.1. The Sensitivity of Antimicrobial Substances from *L. plantarum* LKDS 1.0344 to Proteolytic Enzymes

For the rectification of the antimicrobial elements of *L. plantarum* CFS were prepared by incubating CFS (1 h at 37 °C) with protease, pepsin, bromelain, pronase, α-chymotrypsin, and ficin. All enzymes were utilized in potassium phosphate buffer (10 mmol per L, pH 7.0) with a concentration of 5 mg per mL. Controls were sustained by using CSF in buffer without having enzymes. Residual antimicrobial inhibition of *S. aureus*, *E. coli*, *L. monocytogenes* and, S. *typhimurium* was assessed with the use of Oxford Cups. All experiments were replicated thrice [11].

#### 4.7.2. Thermal Stability of Partially Purified Antimicrobial Compounds

Incubation of partially purified bacteriocin containing samples was carried out at respective temperatures of 80 °C, 100 °C, 120 °C and samples were taken at respective intervals of 20, 30 and 40 min. Samples were cooled to room temperature after heat treatment, and agar well diffusion test was done to assess the bactericidal of bacteriocin [92]. The Inhibition activity (%) was calculated as in Equation (1).

(1) $$\frac{\left[{\left(\mathrm{inhibition}\;\mathrm{zone}\;\mathrm{diameter}\right)}^{2}-{\left(10\mathrm{mm}\right)}^{2}\right]}{\left[{\left(\max.\;\mathrm{inhibition}\;\mathrm{zone}\;\mathrm{diameter}\right)}^{2}-{\left(10\mathrm{mm}\right)}^{2}\right]}\times 100$$

Inhibition zone diameter = mean value of the diameter of the treated sample (mm)

Max. Inhibition zone diameter = mean value of diameter for control (mm)

#### 4.7.3. pH Stability of Partially Purified Antimicrobial Compounds

The pH stability of partially purified bacteriocin was tested by gradual pH adjustment from 2–8, in stages of one pH unit using 1N NaOH or 1N HCl. The incubation of the samples was done for 1 h at 30 °C and agar well diffusion technique was utilized to admeasure the bactericidal activity of the (partially refined) bacteriocin in triplicate against *L. monocytogenes*, *E. coli*, *S. typhimurium* and *S. aureus* were used as indicators. Inhibition activity (%) was calculated by using Equation (1) [67].

### 4.8. Electrophoresis Analysis

The molecular weight of partially purified bacteriocin preparations was determined by SDS–PAGE (Sodium dodecyl sulfate–polyacrylamide gel electrophoresis). The 30 µL sample was homogenized with 2% SDS and dithiothreitol (DTT) adding a little glycerin to increase the density and heating in boiling water for 5 min deal and then loaded onto gel, along with molecular weight marker mix like by using a Mini- Protean 4 cell system (Bio-Rad, Hercules, CA, USA), 100 V and 20 mA in the separation gel at pH 8.3. The gel was stained with 50 mL of (0.1 M) Coomassie brilliant blue R-250 for 4 h. The decolorizing solution was decolorized for 12 h until the electrophoresis band was clear. After the background was decolorized and cleaned, the gel imaging system was used for imaging and data acquisition [91].

### 4.9. Statistical Analyses

Entire analyses were completed in triplicate and the data was compiled. Tukey method was utilized by using the statistical program Statistics 8.1 (Analytical Software, SAS/STAT^®^, Cary, NC, USA) for analysis of variance (ANOVA) and comparison of means. Statistically, the difference was pondered as significant when the *p* < 0.05. Standard errors and mean values were deliberated and demonstrated in charts as coordinate pairs with error bars.

## 5. Conclusions

This study explored that the *L. plantarum* KLDS 1.0344 possesses a substantial antimicrobial capability against miscellaneous pathogenic bacterial strains, including both the Gram negative and Gram positive types *in vitro*. Acid production, as well as the biosynthesis of bacteriocin-like compounds is anticipated as one of the mechanisms through which the antimicrobial activity is wielded by *L. plantarum* KLDS1.0344. Bacteriocin-like compounds cultivated from *L. plantarum* KLDS1.0344 displayed vehement antibacterial competency against foodborne pathogens, pH and heat stability and, sensitivity to proteolytic enzymes. The outcome of the present study provides information about the antimicrobial potentials of the KLDS strains and this information could be helpful to select the probiotics, starters and natural antimicrobial agents against foodborne pathogens in meat-based processed or partially processed food products (Hams, salami, sausages), milk based fermented (cheeses, kefir, yoghurt, sour cream) and non-fermented products (infant formula powders, ice creams, desserts). As in this modern era the consumers are aware of the concepts of food safety, the consumption of healthy and safer food is getting prime importance, so, on the basis of results obtained from the current study, it might be anticipated that these discoveries will intensify the use of *L. palntarum* in the milk and meat-based food processing industries to produce safe, healthy and longer shelf life food products.

## Figures and Tables

**Table 1:** Antimicrobial potential of *L. plantarum* KLDS 1.0317, 1.0318, 1.0344, 1.0386, 1.0628, 1.0985, 1.0986 cultures’ by measuring inhibition zones (mm) against indicator pathogens.

	**KLDS 1.0317**	**KLDS 1.0318**	**KLDS 1.0344**	**KLDS 1.0386**	**KLDS 1.0628**	**KLDS 1.0985**	**KLDS 1.0986**
**pH**	**4.6 ± 0.0 ^E^**	**4.9 ± 0.0 ^C^**	**3.4 ± 0.1 ^G^**	**5.5 ± 0.1 ^A^**	**5.3 ± 0.0 ^B^**	**4.7 ± 0.0 ^D^**	**4.4 ± 0.0 ^F^**
**Pathogens**	**Culture**	**Culture**	**Culture**	**Culture**	**Culture**	**Culture**	**Culture**
***L. monocytogenes***	11.2 ± 0.1 ^aA^	5.6 ± 0.1 ^abE^	9.2 ± 0.1 ^bB^	7.9 ± 0.0 ^aD^	-	8.8 ± 0.1 ^a^^C^	9.3 ± 0.1 ^aB^
***S. aureus***	-	6.9 ± 0.2 ^aC^	15.8 ± 0.1 ^aA^	-	5.7 ± 0.0 ^aB^	-	-
***S. typhimurium***	7.8 ± 0.0 ^bB^	-	14.2 ± 0.0 ^aA^	-	-	4.9 ± 0.2 ^b^^C^	-
***E. coli* O157:H7**	-	-	12.7 ± 0.0 ^abA^	-	-	-	-
	**KLDS 1.0317**	**KLDS 1.0318**	**KLDS 1.0344**	**KLDS 1.0386**	**KLDS 1.0628**	**KLDS 1.0985**	**KLDS 1.0986**
**pH**	**5.6 ± 0.1 ^A^**	**4.8 ± 0.0 ^D^**	**3.3 ± 0.1 ^G^**	**5.4 ± 0.0 ^B^**	**5.3 ± 0.0 ^C^**	**4.7 ± 0.0 ^E^**	**4.3 ± 0.1 ^F^**
***Pathogens***	**CFS**	**CFS**	**CFS**	**CFS**	**CFS**	**CFS**	**CFS**
***L. monocytogenes***	8.4 ± 0.2 ^aA^	2.4 ± 0.0 ^cE^	8.5 ± 0.0 ^bA^	4.6 ± 0.1 ^bD^	-	5.3 ± 0.1 ^aC^	6.3 ± 0.0 ^aB^
***S. aureus***	-	3.7 ± 0.0 ^aC^	13.8 ± 0.0 ^aA^	-	3.0 ± 0.0 ^bB^	-	-
***S. typhimurium***	4.9 ± 0.0 ^bB^	-	12.4 ± 0.1 ^aA^	-	-	3.0 ± 0.9 ^bC^	-
***E. coli* O157:H7**	-	-	10.4 ± 0.1 ^abA^	-	-	-	-

CFS = Cell free supernatant; (-) no inhibition activity; Results of inhibition zones (mm) are given as the mean value of the triplicate trials ± SD (standard deviation); Values in a column with different small superscript letters and in row with capital superscript letters are significantly different (*p* < 0.05).

**Table 2:** Spectrum of antimicrobial activity of culture of *L. plantarum* KLDS 1.0344 and its CFS by quantifying inhibition zones.

Indicator Bacterium	Medium	Temperature	Sensitivity by Culture	Sensitivity by CFS
pH			3.23 ± 0.06	3.19 ± 0.08
*Lactobacillus paracasei* KLDS1.0201	mMRS	37 °C	+	+
*Lactobacillus plantarum* KLDS 1.0628	mMRS	37 °C	+	+
*Lactobacillus helveticus* KLDS 1.9202	mMRS	37 °C	+	+
*Lactobacillus helveticus* KLDS 1.9204	mMRS	37 °C	+	+
*Lactococcus lactis* KLDS 4.0325	M17	37 °C	+	−
*Sterptococcus thermophilus* KLDS 3.0207	M17	37 °C	−	+
*Escherichia coli* ATCC 43889	BHI	37 °C	+++	+++
*Salmonella typhimurium* ATCC 14028	BHI	37 °C	+++	+++
*Staphylococcus aureus* ATCC 25923	BHI	37 °C	+++	+++
*Listeria monocytogenes* ATCC 19115	BHI	37 °C	++	++
*Lactobacillus plantarum* KLDS 1.0986	mMRS	37 °C	−	−

Inhibition zone measurements (mm): No inhibition (−), 1 to 5 mm (+), 5 to 10 mm (++), bigger than 10 mm (+++).

**Table 3:** The effect of proteolytic enzymes on the antibacterial action of the antibacterial peptide from *L. plantarum* KLDS 1.0344 inhibiting the *L. monocytogenes* ATCC 19115, *S. typhimurium* ATCC 14028, *E. coli* O157:H7 ATCC 43889, *S. aureus* ATCC 25923 by measured inhibition zone diameters (mm).

Enzyme	*L. monocytogenes*	*S. aureus*	*S. typhimurium*	*E. coli* O157:H7
Control	15.2 ± 0.6 ^a^	21.7 ± 0.9 ^a^	18.3 ± 0.7 ^a^	17.8 ± 0.7 ^a^
Pepsin	12.7 ± 0.5 ^e^	18.5 ± 0.8 ^d^	16.3 ± 0.7 ^d^	15.6 ± 0.6 ^d^
Protease	14.5 ± 0.5 ^b^	20.4 ± 0.8 ^b^	17.9 ± 0.7 ^ab^	17.2 ± 0.7 ^b^
Pronase	13.3 ± 0.5 ^de^	18.7 ± 0.8 ^cd^	16.8 ± 0.6 ^cd^	16.2 ± 0.6 ^cd^
Bromelain	12.5 ± 0.5 ^e^	18.2 ± 0.7 ^e^	16.0 ± 0.6 ^e^	15.3 ± 0.6 ^e^
Ficin	14.0 ± 0.6 ^c^	19.8 ± 0.8 ^bc^	17.5 ± 0.7 ^b^	16.7 ± 0.7 ^bc^
α-Chymotrypsin	13.5 ± 0.6 ^d^	19.3 ± 0.8 ^c^	17.0 ± 0.6 ^c^	16.4 ± 0.7 ^c^

Data is shown as mean ± SD (standard deviation); The values presented in a column with different superscripts are significantly different (*p* < 0.05).

**Table 4:** Effect of pH on the antibacterial activity of bacteriocins from *L. plantarum* KLDS 1.0344 against *Listeria monocytogenes* ATCC 19115, *Salmonella typhimurium* ATCC 14028, *Escherichia coli* O157:H7 ATCC 43889, *Staphylococcus aureus* ATCC 25923.

pH	*L. monocytogenes*	*S. aureus*	*S. typhimurium*	*E. coli* O157:H7
2	100.0 ± 0.0 ^a^	100.0 ± 0.0 ^a^	100.0 ± 0.0 ^a^	100.0 ± 0.0 ^a^
3	82.3 ± 0.3 ^b^	78.1 ± 0.2 ^b^	73.2 ± 0.3 ^b^	71.4 ± 0.2 ^b^
4	65.4 ± 0.2 ^d^	60.0 ± 0.2 ^c^	56.4 ± 0.2 ^c^	54.0 ± 0.2 ^c^
5	50.0 ± 0.2 ^e^	42.4 ± 0.1 ^d^	39.1 ± 0.1 ^d^	33.0 ± 0.1 ^d^
6	29.2 ± 0.1 ^f^	20.2 ± 0.1 ^f^	16.0 ± 0.0 ^f^	15.2 ± 0.0 ^f^
7	0.0 ± 0.0 ^g^	0.0 ± 0.0 ^g^	0.0 ± 0.0 ^g^	0.0 ± 0.0 ^g^
8	0.0 ± 0.0 ^g^	0.0 ± 0.0 ^g^	0.0 ± 0.0 ^g^	0.0 ± 0.0 ^g^

Inhibition activity (%) = [(diameter of inhibition zone)^2^ − (10 mm)^2^/(diameter of maximum inhibition zone)^2^ − (10 mm)^2^] × 100%. The data values are expressed as the mean ± standard deviation (*n* = 3). Values in a column with different superscript letters are significantly different (*p* < 0.05).

**Table 5:** Effect of heat on the antibacterial action of bacteriocins from *L. plantarum* KLDS 1.0344 encountering *L. monocytogenes* ATCC 19115, *S. typhimurium* ATCC 14028, *E. coli* O157:H7 ATCC 43889, *S. aureus* ATCC 25923. All results are presented in inhibition activity (%).

Temperature/Time	*L. monocytogenes*	*S. aureus*	*S. typhimurium*	*E. coli* O157:H7
80/20	100.0 ± 0.0 ^a^	100.0 ± 0.0 ^a^	100.0 ± 0.0 ^a^	100.0 ± 0.0 ^a^
80/30	100.0 ± 0.0 ^a^	100.0 ± 0.0 ^a^	100.0 ± 0.0 ^a^	100.0 ± 0.0 ^a^
80/40	100.0 ± 0.0 ^a^	100.0 ± 0.0 ^a^	100.0 ± 0.0 ^a^	100.0 ± 0.0 ^a^
100/20	100.0 ± 0.0 ^a^	100.0 ± 0.0 ^a^	100.0 ± 0.0 ^a^	100.0 ± 0.0 ^a^
100/30	100.0 ± 0.0 ^a^	100.0 ± 0.0 ^a^	100.0 ± 0.0 ^a^	100.0 ± 0.0 ^a^
100/40	100.0 ± 0.0 ^a^	100.0 ± 0.0 ^a^	100.0 ± 0.0 ^a^	100.0 ± 0.0 ^a^
120/20	85.0 ± 0.7 ^b^	86.5 ± 0.3 ^b^	83.0 ± 0.1 ^b^	81.0 ± 0.2 ^b^
120/30	74.3 ± 0.4 ^c^	78.0 ± 0.2 ^c^	71.5 ± 0.1 ^c^	69.0 ± 0.0 ^c^
120/40	61.2 ± 0.1 ^d^	54.5 ± 0.1 ^d^	47.0 ± 0.3 ^d^	40.5 ± 0.0 ^d^

Inhibition activity (%) = [(diameter of inhibition zone)^2^ − (10 mm)^2^/(diameter of maximum inhibition zone)^2^ − (10 mm)^2^] × 100%. The data values are expressed as the mean ± SD (*n* = 3). Values in a column with different superscripts are significantly different (*p* < 0.05).
